# Supplementary material for: Empirical methods for controlling false positives and estimating confidence in ChIP-Seq peaks
Source: BMC Bioinformatics. 2008 Dec 5;9:523. doi: 10.1186/1471-2105-9-523 (PMC2628906; doi:10.1186/1471-2105-9-523)
Supplement: Additional File 1 — A variety of html documents from the USeq web site detailing the available applications, their best usage, output file type descriptions, command line menus, etc. [file 1471-2105-9-523-S1.zip › USeqUserGuides/JavaDoc.zip!/JavaDoc/edu/utah/seq/methylation454/Read.html]

Read


|  |  |  |
| --- | --- | --- |
| **Overview** \| **Package** \| **Class** \| **Use** \| **Tree** \| **Deprecated** \| **Index** \| **Help** | |  |
| **PREV CLASS**   **NEXT CLASS** | **FRAMES**    **NO FRAMES**     **All Classes** |  |
| SUMMARY: NESTED \| FIELD \| CONSTR \| METHOD | DETAIL: FIELD \| CONSTR \| METHOD |  |


---


## edu.utah.seq.methylation454 Class Read

```
java.lang.Object
  edu.utah.seq.methylation454.Read
```

---

``` public class Read extends java.lang.Object ```

Sequence aligned to a reference.

---

**Constructor Summary**

`Read(java.lang.String header,
java.lang.String seqLine)`
  
          example of a header and a seqLine
>ETK72AO01CGP6K /amp="I14" /cId="CON\_1" /id="ETK72AO01CGP6K" /qCd="24-45" /lCp="5" /rCd="24-45" /rRg="24-56" /mFc="60" /str="1"
23 AA-T-AA-T-AAA-AA-TTTT-A-G-A-TA-TG 1452


| **Method Summary** | |
| --- | --- |
| `int` | `getAlignmentEnd()` |
| `int` | `getAlignmentStart()` |
| `java.lang.String` | `getSequence()` |
| `java.lang.String` | `getStrand()` |
| `void` | `setAlignmentEnd(int alignmentEnd)` |
| `void` | `setAlignmentStart(int alignmentStart)` |
| `void` | `setSequence(java.lang.String sequence)` |
| `void` | `setStrand(java.lang.String strand)` |

**Methods inherited from class java.lang.Object**

`equals, getClass, hashCode, notify, notifyAll, toString, wait, wait, wait`

**Constructor Detail**

### Read

```
public Read(java.lang.String header,
            java.lang.String seqLine)
```

:   example of a header and a seqLine
    >ETK72AO01CGP6K /amp="I14" /cId="CON\_1" /id="ETK72AO01CGP6K" /qCd="24-45" /lCp="5" /rCd="24-45" /rRg="24-56" /mFc="60" /str="1"
    23 AA-T-AA-T-AAA-AA-TTTT-A-G-A-TA-TG 1452


**Method Detail**

### getAlignmentEnd

```
public int getAlignmentEnd()
```

---


### setAlignmentEnd

```
public void setAlignmentEnd(int alignmentEnd)
```

---


### getAlignmentStart

```
public int getAlignmentStart()
```

---


### setAlignmentStart

```
public void setAlignmentStart(int alignmentStart)
```

---


### getSequence

```
public java.lang.String getSequence()
```

---


### setSequence

```
public void setSequence(java.lang.String sequence)
```

---


### getStrand

```
public java.lang.String getStrand()
```

---


### setStrand

```
public void setStrand(java.lang.String strand)
```


---


|  |  |  |
| --- | --- | --- |
| **Overview** \| **Package** \| **Class** \| **Use** \| **Tree** \| **Deprecated** \| **Index** \| **Help** | |  |
| **PREV CLASS**   **NEXT CLASS** | **FRAMES**    **NO FRAMES**     **All Classes** |  |
| SUMMARY: NESTED \| FIELD \| CONSTR \| METHOD | DETAIL: FIELD \| CONSTR \| METHOD |  |


---
